# Hepatocellular carcinoma: radiomics nomogram on gadoxetic acid-enhanced MR imaging for early postoperative recurrence prediction

**DOI:** 10.1186/s40644-019-0209-5

**Published:** 2019-05-14

**Authors:** Zhen Zhang, Hanyu Jiang, Jie Chen, Yi Wei, Likun Cao, Zheng Ye, Xin Li, Ling Ma, Bin Song

**Affiliations:** 1 Department of Radiology, West China Hospital of Sichuan University, No.37 Guo Xue Xiang, Chengdu, 610041 China; 2 GE Healthcare China, Beijing, China

**Keywords:** Gadoxetic acid-enhanced MRI, Hepatocellular carcinoma, Recurrence, Radiomics, Nomogram

## Abstract

**Background:**

This study was performed to prospectively develop and validate a radiomics nomogram for predicting postoperative early recurrence (≤1 year) of hepatocellular carcinoma (HCC) using whole-lesion radiomics features on preoperative gadoxetic acid-enhanced magnetic resonance (MR) images.

**Methods:**

In total, 155 patients (training cohort: *n* = 108; validation cohort: *n* = 47) with surgically confirmed HCC were enrolled in this IRB-approved prospective study. Three-dimensional whole-lesion regions of interest were manually delineated along the tumour margins on multi-sequence MR images. Radiomics features were generated and selected to build a radiomics score using the least absolute shrinkage and selection operator (LASSO) method. Clinical characteristics and qualitative imaging features were identified by two independent radiologists and combined to establish a clinical-radiological nomogram. A radiomics nomogram comprising the radiomics score and clinical-radiological risk factors was constructed based on multivariable logistic regression analysis. Diagnostic performance and clinical usefulness were measured by receiver operation characteristic (ROC) and decision curves.

**Results:**

In total, 14 radiomics features were selected to construct the radiomics score. For the clinical-radiological nomogram, the alpha-fetoprotein (AFP) level, gross vascular invasion and non-smooth tumour margin were included. The radiomics nomogram integrating the radiomics score with clinical-radiological risk factors showed better discriminative performance (AUC = 0.844, 95%CI, 0.769 to 0.919) than the clinical-radiological nomogram (AUC = 0.796, 95%CI, 0.712 to 0.881; *P* = 0.045), with increased clinical usefulness confirmed using a decision curve analysis.

**Conclusions:**

Incorporating multiple predictive factors, the radiomics nomogram demonstrated great potential in the preoperative prediction of early HCC recurrence after surgery.

**Electronic supplementary material:**

The online version of this article (10.1186/s40644-019-0209-5) contains supplementary material, which is available to authorized users.

## Introduction

Hepatocellular carcinoma (HCC) has become the second most common cancer and ranks as the sixth most common cause of cancer-related death worldwide [1]. Despite the fact that hepatic resection remains the main curative treatment for HCC, the high incidence of recurrence poses a major challenge in HCC management [2, 3]. Most of the cases of postoperative recurrence occur in the remnant liver as intrahepatic recurrence, which can be further classified as early or late recurrence [4]. Of these, early recurrence accounts for more than 70% of tumour recurrence and is associated with a worse prognosis [5, 6]. Identifying reliable predictors of early recurrence is crucial for patient risk stratification, treatment decision-support and long-term survival improvement.

Tumour factors, such as multifocality, poor tumour differentiation and microvascular invasion, have been identified as risk factors for early recurrence [7–9]. However, most of these factors can only be evaluated postoperatively with histopathologic examination. Recently, studies on magnetic resonance (MR) imaging, particularly with the hepatocyte specific contrast agent gadoxetic acid (formerly known as Gd-EOB-DTPA, Bayer Healthcare, Germany), have reported several qualitative imaging features, such as peritumoural parenchymal enhancement, satellite nodules and non-smooth tumour margin, to be non-invasive predictors of early recurrence in HCC [10–12]. However, these criteria for preoperative imaging prediction of early recurrence in HCC have not yet been widely recognized.

Radiomics enables an in-depth characterization of tumour phenotypes by converting traditional medical images into high-dimensional, quantitative and mineable imaging data and has demonstrated potential in providing intratumour information on heterogeneous tumours and predicting posttreatment survival in the field of oncology [13, 14]. For HCC, preliminary evidence has suggested that radiomics features were potentially predictive of overall survival, tumour recurrence and treatment response [15–17]. Hui et al. suggested that single radiomics features derived from MR images may be able to predict early HCC recurrence [18]. However, most of the published studies were retrospective and lacked an independent validation cohort to assess the generalizability and reproducibility of the results. In addition, combined analysis of multiple relevant predictors from radiomics features, clinical data and qualitative imaging features remains limited.

Therefore, the aim of this prospective study was to investigate the predictive value of the radiomics features extracted from gadoxetic acid-enhanced MR images. Furthermore, a radiomics nomogram, comprising radiomics features and clinical-radiological factors, was developed and validated for predicting the early recurrence of HCC following hepatectomy.

## Materials and methods

### Patients

**Fig. 1:**
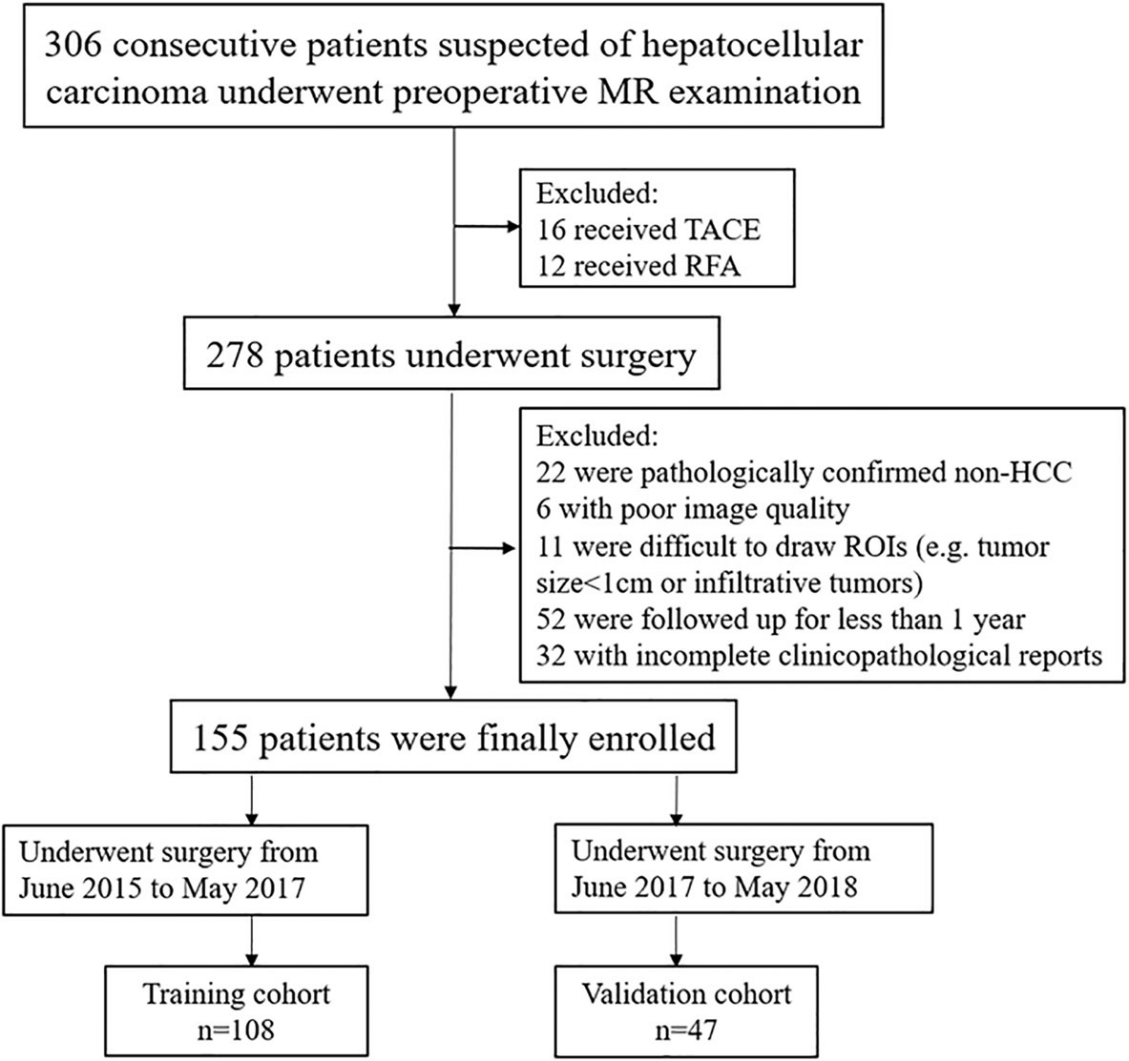
Patient recruitment process

Institutional review board ethical approval and informed consent from all patients were acquired before patient enrolment. From June 2015 to May 2018, 306 consecutive patients with suspected primary liver lesions based on clinical history or previous ultrasonography or CT results underwent preoperative gadoxetic acid-enhanced MR imaging in our hospital. 28 patients receiving other treatment, including trans-arterial chemoembolization (*n* = 16) and radiofrequency ablation (*n* = 12), were initially excluded (Fig. 1). Inclusion criteria included 1) patient age > 18 years old; 2) primary liver lesions without prior treatment; 3) patients without contraindications for gadoxetic acid-enhanced MR examination; and 4) hepatectomy within 7 days after the MR imaging examination. In total, 123 patients were excluded for the following reasons: 1) lesions pathologically confirmed as non-HCC (*n* = 22); 2) cases with poor image quality and motion artefacts (*n* = 6); 3) small HCC lesions (size < 1 cm) (*n* = 3) and ruptured or infiltrative tumours (*n* = 8) for which it was difficult to draw regions of interests (ROIs); 4) cases with a follow up period less than 1 year (*n* = 52); and 5) incomplete clinicopathological data (*n* = 32).

Figure 1 illustrates the flow chart for the study population. For analysis, enrolled patients who underwent surgery between June 2015 and May 2017 were assigned to the training cohort used to construct nomograms; the subsequent patients who underwent surgery from June 2017 to May 2018 were assigned to the validation cohort.

Demographic and clinicopathologic data were completely recorded for all patients (Table 1).

**Table 1:** Patient characteristics in the training and validation cohort

Variables	Training cohort (n = 108)	Validation cohort (n = 47)	P
Clinical characteristics			
Age, mean ± SD, years	50.06 ± 11.44	51.02 ± 11.96	0.159
Gender			0.257
Female	22	9	
Male	86	38	
Recurrence rate (%)	48.15	48.94	0.474
Child-Pugh score			0.000
A	107	47	
B	1	0	
Barcelona Clinic Liver Cancer (BCLC) stage			0.146
0	8 (7.5%)	5 (10.6%)	
A	26 (24.0%)	8 (17.0%)	
B	49 (45.4%)	19 (40.4%)	
C	25 (23.1%)	15 (32.0%)	
AFP (ng/ml)			0.999
≤ 400	60	26	
> 400	48	21	
CEA (ng/ml)			0.832
≤ 3.4	85	40	
> 3.4	23	7	
CA19–9 (ng/ml)			0.967
≤ 22	62	29	
> 22	46	18	
HBV-DNA (IU/ml)			0.778
≤ 100	44	15	
> 100	64	32	
HBsAg			0.999
Positive	92	40	
Negative	16	7	
ALT (IU/l)			0.988
≤ 50	61	27	
> 50	47	20	
AST (IU/l)			0.850
≤ 35	53	22	
> 35	55	25	
TBIL (μmol/l)			0.639
≤ 17.1	100	46	
> 17.1	8	1	
DBIL (μmol/l)			0.997
≤ 8.8	83	36	
> 8.8	25	11	
IBIL (μmol/l)			0.996
≤ 20	102	45	
> 20	6	2	
ALB (g/l)			0.611
< 45	90	43	
> 45	18	4	
PT (s)			0.179
< 9.6 or > 12.8	29	11	
9.6–12.8	79	36	
PLT (× 10^9/l)			0.991
≤ 100	85	38	
> 100	23	9	
Qualitative imaging findings			
Signal on HBP images, mean ± SD	211.23 ± 82.49	182.95 ± 77.87	0.051
Tumour size			0.267
≤ 5 cm	53 (49.1%)	15 (31.9%)	
> 5 cm	55 (50.9%)	32 (68.1%)	
Multifocality			0.816
Present	35 (32.4%)	19 (40.4%)	
Absent	73 (67.6%)	28 (59.6%)	
Tumour margin			0.999
Smooth	43 (39.8%)	19 (40.4%)	
Non-smooth	65 (60.2%)	28 (59.6%)	
Gross vascular invasion			0.117
Present	37 (34.3%)	25 (53.2%)	
Absent	71 (65.7%)	22 (46.8%)	
Radiologic capsule			0.839
Present	80 (74.1%)	38 (80.8%)	
Absent	28 (25.9%)	9 (19.2%)	
Peritumoural enhancement			0.978
Present	51 (47.2%)	21 (44.7%)	
Absent	55 (52.8%)	26 (55.3%)	
Peritumoural hypointensity on HBP images			0.999
Present	61 (56.5%)	27 (57.5%)	
Absent	47 (43.5%)	20 (42.5%)	

*P* values were obtained from the univariate regression analyses between the training cohort and the validation cohort

*AFP* alpha-fetoprotein, *CEA* carcinoembryonic antigen, *HBV-DNA* hepatitis B virus DNA load, *HBsAg* hepatitis B surface antigen status, *ALT* alanine aminotransferase, *AST* aspartate aminotransferase, *TBIL* total bilirubin, *DBIL* direct bilirubin, *IBIL* indirect bilirubin, *ALB* albumin, *PT* prothrombin time, *PLT* platelet count, *SD* standard deviation

### Follow-up

Monthly blood tests for serum alpha-fetoprotein (AFP) level and contrast-enhanced CT or gadoxetic acid-enhanced MR imaging were conducted during follow-up the first month after surgery and every 3 months thereafter. Early recurrence was diagnosed by the combined findings of these clinical examinations and defined as intrahepatic and/or extrahepatic recurrence within 1 year after HCC tumour resection.

### MR techniques

All MR scans were performed on a 3.0 T MR scanner (Magnetom Skyra, Siemens Healthcare, Erlangen, Germany) with an 18-channel body array coil. All patients were instructed to fast for 6–8 h before the scan. The routine MR sequences included breath-hold fat-suppressed fast spin-echo T2-weighted imaging, MR cholangiopancreatography (MRCP) heavily T2-weighted 2D imaging, and a diffusion-weighted sequence (b values: 0, 50, 500, 800, 1000, and 1200 s/mm2). For gadoxetic acid-enhanced imaging (Primovist®; Bayer Schering Pharma AG, Berlin, Germany), the unenhanced phase, arterial phase (20–35 s), portal venous phase (60–70 s), transitional phase (3 min) and hepatobiliary phase (HBP, 20 min) were obtained using a fat-suppressed 3D gradient-echo T1 weighted sequence (volume interpolated breath-hold examination, VIBE). The contrast agent was administered intravenously at a dose of 0.025 mmol/kg at a rate of 2 ml/s and followed immediately by a 30-ml saline flush. Detailed MR imaging sequences and parameters are provided in Additional file 1: Table S1.

### Imaging analysis

#### Qualitative imaging features

All images were derived from a picture archiving and communication system in Digital Imaging and Communications in Medicine (DICOM) format. Two independent radiologists (reader 1 and reader 2, with 6 and 10 years of experience in abdominal imaging, respectively), who were blinded to all clinicopathologic results and imaging interpretations of the other reader, evaluated the following imaging features: (1) tumour size (the maximum diameter measured on portal venous phase images); (2) multifocality (defined as more than three nodules on portal venous phase images); (3) tumour margin (smooth or non-smooth); (4) radiologic capsule (defined as a smooth, uniform, and sharply outlined area of enhancement around the tumour margin on portal venous phase images); (5) peritumoural enhancement (defined as peritumoural enhancement in the late arterial phase or early portal venous phase); (6) peritumoural hypointensity on HBP images (defined as decreased signal intensity, in whole or in part, around the tumour compared with the adjacent liver parenchyma); (7) gross vascular invasion (defined as invasion of the adjacent hepatic arteries or hepatic veins grossly visible on images) [10]; and (8) signal intensity on HBP images. Disagreements regarding the presence or absence of the qualitative radiologic features were resolved by consensus.

#### Tumour segmentation and radiomics feature extraction

**Fig. 2:**
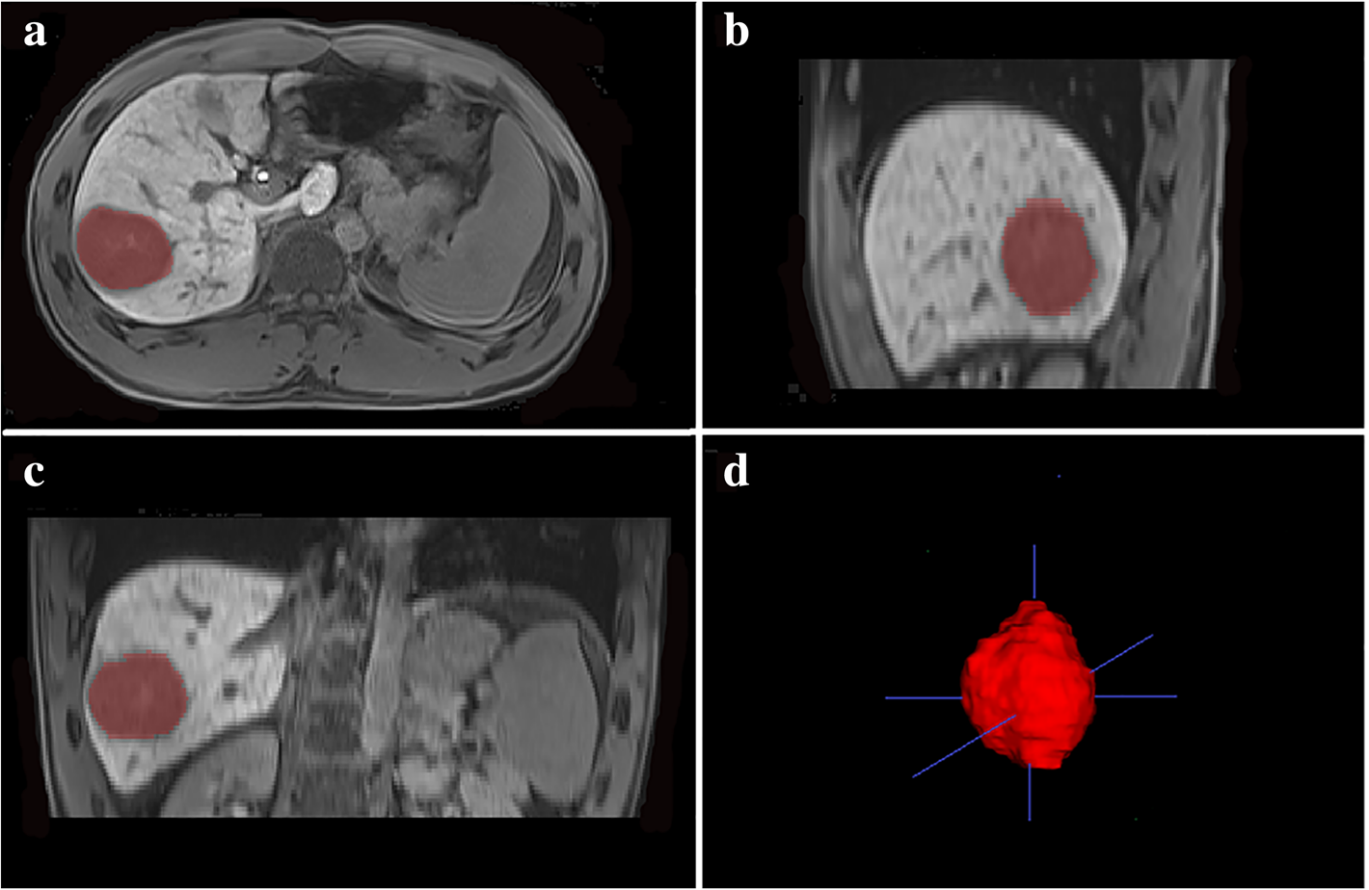
A 47-year-old male with histologically confirmed HCC. **(a, b, c)** Representative manual segmentation of the whole lesion in the hepatobiliary phase illustrated on three planes. The dotted lines represent the delineations of the ROIs used to derive the radiomics features. **(d)** Three-dimensional (3D) volumetric reconstruction of the segmented lesion

T2-weighted imaging, unenhanced T1-weighted imaging, gadoxetic acid-enhanced arterial phase, portal venous phase and HBP images were used for feature extraction. ITK-SNAP software (version 3.6.0, open source, http://www.itksnap.org/pmwiki/pmwiki.php) was used for whole-tumour three-dimensional (3D) segmentation. ROIs were manually delineated around the boundary of the entire tumour on each slice by an experienced radiologist with 6 years of experience in abdominal imaging (Fig. 2). Another independent radiologist with 4 years of experience repeated the same procedure of tumour segmentation within 1–2 weeks to calculate the intraclass correlation coefficients (ICCs). Finally, 385 radiomics features were generated from each MR sequence using an in-house scientific research 3D analysis software (Analysis-Kit, version V3.0.0.R, GE healthcare), including the following categories: histogram, texture features, form factors, grey-level co-occurrence matrix (GLCM), and grey-level run-length matrix (GLRLM).

### Statistical analysis

A least absolute shrinkage and selection operator (LASSO) logistic regression algorithm was performed to select the most predictive radiomics features, with a 10-fold cross validation applied for overfitting reduction [19]. A radiomics score was calculated for each patient based on a linear combination of the selected radiomics features weighted by their LASSO coefficients. Inter-observer ICCs were used to measure the inter-observer agreement of radiomics feature extraction. Interobserver agreement of MR imaging feature evaluation was qualified by Cohen’s k statistic [20].

Univariate and multivariate logistic regression analyses were performed in the training cohort to identify the independent risk factors for early recurrence. Significant variables with *P* < 0.05 in the univariate analysis were included in the multivariate logistic regression analysis to identify the independent risk factors for the radiomics nomogram construction. The clinical-radiological nomogram was constructed with only the clinical-radiological risk factors. Odds ratios (ORs), as estimates of relative risk, with 95% confidence intervals (CIs) were obtained for each risk factor, and the goodness-of-fit for logistic regression was evaluated with the Hosmer-Lemeshow test [21].

The discriminative performances of the nomograms were quantified by the area under the curve (AUC) of receiver operator characteristic (ROC) curves. Differences in the ROC curves were compared by using the Delong test. Calibration curves were generated to assess the calibration of the nomograms. The probabilities of net benefits were quantified by decision curve analysis (DCA) to evaluate the clinical application value of the nomograms [22].

The statistical analyses were implemented using R statistical software (version 3.4.2, *http://www.R-project.org*), and two-sided *P* values < 0.05 were considered significant. The packages in R that were used in this study are listed in Additional file 1: Part 3.

## Results

### Patients characteristics

In total, 155 patients (male/female: 124/31; mean age, 50.35 ± 11.57 years, range 26 to 77 years) were included in the final study group and divided into the training cohort (*n* = 108) and validation cohort (*n* = 47). Among the included patients, early recurrence was identified in 75 (48.3%) patients (intrahepatic recurrence: *n* = 58; extrahepatic recurrence: *n* = 8; concurrent intra- and extrahepatic recurrence: *n* = 9) (Table 1). The median time to recurrence was 6.75 months (range, 3–11 months). There was no difference in the early recurrence rate between the training cohort (52/108, 48.1%) and the validation cohort (23/47, 48.9%, *P* = 0.474).

Demographic and clinicopathological characteristics of the included patients are summarized in Table 1. No significant difference was detected for any characteristic between the training and validation cohorts (*P* = 0.146 to 0.996).

### Development of radiomics score

In total, 13 radiomics features with non-zero coefficients (2 features from T2-weighted images, 6 features from arterial phase images and 5 features from HBP images) were selected to calculate the radiomics score using the formula described in Additional file 1: Table S2. The name and description of the selected features can be found in Additional file 1: Part 2b. The agreement upon selected radiomics features between the two radiologists were substantial to excellent (ICC range: 0.642 to 0.948).

### Development of predictive nomograms

**Fig. 3:**
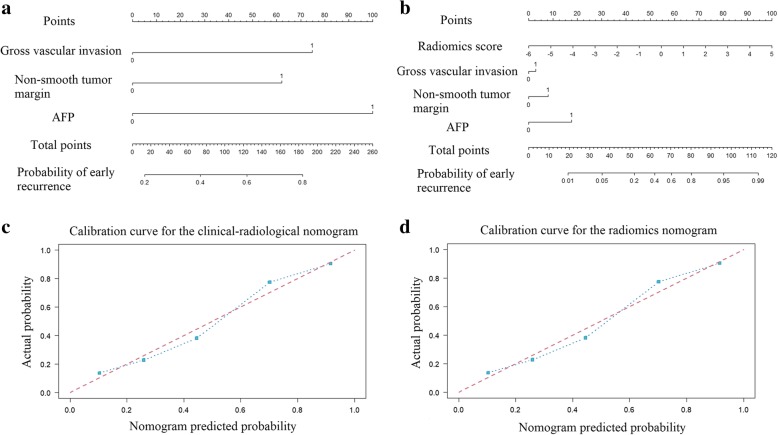
The developed clinical-radiological nomogram **(a)** and radiomics nomogram **(b)**. From each variable location on the corresponding axis, draw a line straight upward to the point axis and obtain a point. After adding up all the points, draw a line from the total points axis to the bottom line to determine the probability of developing early recurrence. Calibration curves for the clinical-radiological nomogram **(c)** and radiomics nomogram **(d)** in the training cohort (Hosmer-Lemeshow test; *P = 0.145 and 0.214*, respectively). The actual outcome of early recurrence is represented on the y-axis, and the predicted probability is represented on the x-axis. The closer fit of the diagonal blue line to the ideal red line indicates the predictive accuracy of the nomogram

In total, 1 clinical characteristic (AFP level), 6 qualitative imaging features (tumour size, multifocality, non-smooth tumour margin, gross vascular invasion, peritumoural enhancement, and peritumoural hypointensity on HBP images) and the radiomics score were identified by univariate analysis (all *P < 0.05*). In the multivariable logistic regression analysis, the radiomics score (OR, 2.433 [95%CI, 1.436 to 4.473], *P = 0.002*), the AFP level (OR, 2.112 [95%CI, 0.488 to 9.974], *P < 0.001*), gross vascular invasion (OR, 3.356 [95%CI, 1.308 to 9.023], *P = 0.013*) and a non-smooth tumour margin (OR, 2.735 [95%CI, 1.104 to 6.989], *P = 0.031*) significantly predicted early recurrence (Table 2). Thus, the clinical-radiological nomogram was constructed including the AFP level, gross vascular invasion and non-smooth tumour margin (Fig. 3a). The radiomics nomogram was constructed with the radiomics score, AFP level, gross vascular invasion and non-smooth tumour margin (Fig. 3b).

**Table 2:** Univariate and multivariate regression analyses between early recurrence and non-recurrence groups in the training cohort

Intercept and variables	Univariate analysis			Multivariate analysis		
	β	Odd ratios (95%CI)	P	β	Odd ratios (95%CI)	P
Intercept	–	–	–	1.599	–	< 0.001*
AFP level	1.918	6.809 (2.984–16.419)	0.000*	1.621	2.112 (0.488–9.974)	< 0.001*
Tumour size	0.905	2.472 (1.149–5.438)	0.021*	–	–	–
Multifocality	1.067	2.908 (1.274–6.912)	0.012*	–	–	–
Gross vascular invasion	1.408	4.090 (1.778–9.923)	0.001*	1.211	3.356 (1.308–9.023)	0.013*
Non-smooth tumour margin	1.159	3.189 (1.446–7.271)	0.004*	1.006	2.735 (1.104–6.989)	0.031*
Peritumoural hypointensity on HBP images	0.865	2.376 (1.097–5.271)	0.036*	–	–	–
Peritumoural enhancement	1.136	3.116 (1.436–6.955)	0.004*	–	–	–
Radiomics score	1.000	2.718 (1.691–4.370)	0.000*	0.889	2.433 (1.436–4.473)	0.002*

Significant variables with *P* < 0.05 in the univariate analysis were included in the multivariate logistic regression analysis

*AFP* alpha-fetoprotein, *CI* confidence internal

β is the regression coefficient. * *P* value < 0.05

Interobserver agreement on the identified MR imaging features were substantial to excellent (had *k* values of *0.910* for gross vascular invasion and *0.793* for non-smooth tumour margin).

### Predictive performance of the nomograms

In the training cohort, the AUCs of the radiomics score, clinical-radiological nomogram and radiomics nomogram were 0.757 (95% CI, 0.667 to 0.846), 0.796 (95% CI, 0.712 to 0.881) and 0.844 (95% CI, 0.769 to 0.919), respectively. The radiomics nomogram demonstrated a significantly higher AUC than the radiomics score (*P = 0.012*) and the clinical-radiological nomogram (*P = 0.045*). The radiomics score and clinical-radiological nomogram showed comparable discriminative power in the training cohort (AUC, 0.757 vs. 0.796, *P = 0.453*) and the validation cohort (AUC, 0.728 vs. 0.814, *P = 0.310*). The diagnostic performance of the radiomics score and two nomograms is shown in Table 3.

**Table 3:** Predictive performance of the three models

	Training cohort				Validation cohort			
	AUC(95%CI)	SEN	SPE	P	AUC(95%CI)	SEN	SPE	P
(1) Radiomics score	0.757 (0.667–0.846)	92.3	44.6		0.728 (0.580–0.877)	69.6	70.8	
(2) Clinical-radiological nomogram	0.796 (0.712–0.881)	75.0	76.8		0.814 (0.682–0.947)	78.3	83.3	
(3) Radiomics nomogram	0.844 (0.769–0.919)	73.1	85.7		0.841 (0.722–0.959)	91.3	75.0	
1 vs. 2				0.453				0.310
1 vs. 3				0.012*				0.013*
2 vs. 3				0.045*				0.131

1 indicates radiomics score; 2 indicates clinical-radiological nomogram; 3 indicates radiomics nomogram

*SEN* sensitivity, *SPE* specificity, *AUC* area under the curve, *CI* confidence interval

*P < 0.05 indicates a significant difference

**Fig. 4:**
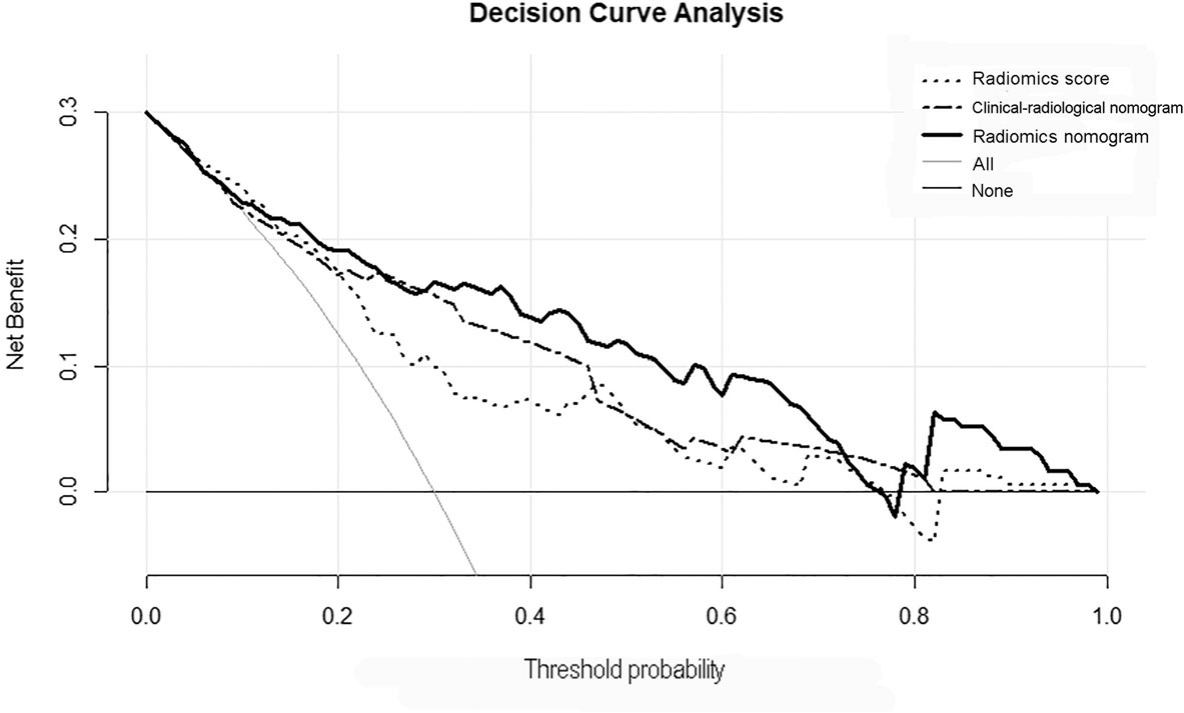
Decision curve analysis for each model. The y-axis measures the net benefit, and the x-axis is the threshold probability. Using the radiomics nomogram for early recurrence prediction has more benefit than either the treat-all-patients scheme (gray line) or the treat-none scheme (horizontal black line). The radiomics nomogram (green line) received a higher net benefit than either the clinical-radiological nomogram or the radiomics score alone across the full range of reasonable threshold probabilities

Acceptable calibrations of the clinical-radiological nomogram and radiomics nomogram are shown in Fig. 3c and d. The Hosmer-Lemeshow test yielded non-significant results for the two nomograms in the training and validation cohorts (all *P > 0.05*). The DCA curve showed that the radiomics nomogram had the largest overall net benefit, compared with the treat-all-patients and treat-none scheme across the full range of reasonable threshold probabilities (Fig. 4).

## Discussion

In this prospective study, a radiomics nomogram incorporating the qualitative imaging features, clinical characteristics and radiomics features derived from gadoxetic acid-enhanced MRI for predicting postoperative early recurrence of HCC was generated. The nomogram demonstrated good discrimination and calibration; thus, it may act as a noninvasive and effective tool to preoperatively identify patients at higher risk for early recurrence following hepatectomy.

Radiomics focuses on improvement of image analysis by extracting hundreds of quantitative features with a computer algorithm and, expectedly, improves the predictive performance of medical images [23]. In the present study, 13 radiomics features closely associated with early recurrence of HCC were extracted from whole-tumour MR images and selected for radiomics score construction. These features are composed of specific categories: three histogram-based features (skewness, voxel value sum and minimum intensity), one texture-based feature (Cluster Prominence), seven GLCM-based features (3 of Correlation, 2 of Inertia, 1 of Energy, and 1 of Inverse Difference Moment) and two GLRLM-based features (2 of High Grey-Level Run Emphasis). Although these features could reflect different aspects of textural information and the underlying tumour biology [24], the correlation between a single radiomics feature and the biological behaviour is difficult to grasp intuitively; instead, construction of multi-feature panels is a more common approach for outcome estimation [25, 26]. We further integrated all these features into a single radiomics score to comprehensively leverage all available information. In our study, the radiomics score demonstrated satisfactory discriminative power both in the training and validation cohorts (AUC = 0.757 and 0.728, respectively), with a markedly high sensitivity of 92.3% in the training cohort, and was indicated to be an independent predictor for early recurrence in the radiomics nomogram (*P = 0.002*). Two recent studies investigated the prognostic value of CT radiomics features for overall survival and recurrence prediction in HCC patients, showing poor to moderate diagnostic performance with AUC values from 0.639 to 0.742 [16, 27]. The main reasons for the better performance of our radiomics score compared with previous CT studies may due to the following: 1) MR imaging may provide better tissue contrast and is less influenced by the artefacts from radiation and bone beam hardening compared to CT images; 2) the use of a hepatobiliary-specific MRI contrast agent (gadoxetic acid) enables the inclusion of radiomics features from multiple phases and facilitates radiomics analysis by providing accurate discrimination of tumour boundaries for segmentation [28, 29]; and 3) 3D ROIs of the whole-tumour were analysed in our study, which can provide more effective and comprehensive evaluation of the entire lesion than the 2D analyses used in previous studies. In addition, one recent study by Hui et al. suggested that single radiomics features derived from MR images may be able to predict early HCC recurrence, with accuracy values ranging from 0.78 to 0.84 [18]. However, their study was retrospective and lacked independent validation. Developed from a prospective cohort, our results indicated that the radiomics score based on gadoxetic acid-enhanced MR images could identify more than 92.3% of early recurrence cases correctly in the training cohort and was well-validated to serve as a quantitative multiple-features parameter for recurrence risk stratification in HCC patients.

Vascular invasion, classified as gross or microvascular invasion, is the expression of the invasive biological behaviour of the tumour and is always associated with a worse clinical outcome [30]. Our study demonstrated that gross vascular invasion was an independent risk factor for early recurrence. This finding was consistent with a previous study, which showed that patients with gross vascular invasion had an increased risk for recurrence after resection [31]. In addition, non-smooth tumour margin was another significant predictor for early recurrence in our study, which was consistent with the results from previous studies [12, 29, 32, 33]. Although the tumour size, multifocality, peritumoural enhancement, and peritumoural hypointensity on HBP images were found significant in the univariate analysis in our study, they lost their statistically significant association with early recurrence in the multivariate analysis. These findings were different from those of previous studies [10]. Such inconsistencies might be associated with the presence of selection bias due to the strict inclusion criteria in our radiomics analysis.

In this study, we further combined the radiomics score with clinical characteristics and subjective imaging features to improve the predictive power of the clinical-radiological nomogram. As a result, the proposed radiomics nomogram yielded an improved diagnostic performance in the training cohort (AUC from 0.796 to 0.844) and validation cohort (AUC from 0.814 to 0.841), indicating that the combined radiomics approach may have a greater value in postoperative early recurrence prediction over the clinical-radiological features. This finding was consistent with Zhou Y et al. [34], who also integrated CT-based radiomics features in conjunction with clinical risk factors and obtained better performance than the use of clinical risk factors alone in predicting early recurrence in HCC.

Moreover, the decision curve analysis showed that our radiomics nomogram demonstrated great potential for clinical application in postoperative outcome estimation. With the help of this radiomics nomogram, patients can be risk-stratified in terms of follow-up. This proposed radiomics nomogram may assist clinicians in evaluating the probability of early recurrence after resection, extend the existing criteria for surgical candidate selection and optimize treatment decisions before patients undergo operation. For instance, patients identified as high risk may benefit from liver transplantation rather than surgical resection; if liver transplantation is not possible, additional adjuvant therapies, such as preoperative adjuvant systemic chemotherapy or upfront transarterial chemoembolization, should be considered preoperatively [35, 36]. Furthermore, even when HCC is removed by curative liver resection, high risk patients should be carefully monitored to detect recurrent HCC at its earliest stage, and appropriate postoperative adjuvant therapy is also required to prevent recurrence after resection in this population [37, 38].

However, the current study has several limitations. 1) Our study was a prospective longitudinal cohort study; thus, the sample size of our study was limited by the strict inclusion criteria. 2) Our study was performed in a single institution. Although all MR images in this prospective study were acquired in a uniform MR scanner with standardized sequences for imaging acquisition to reduce bias and variance of our results, additional validation from other institutions is warranted to facilitate wider use of this predictive nomogram. 3) The complex correlation between a radiomics feature and biological behaviour is difficult to interpret. Further exploration in radio-genomics is still required to determine potential radiomics-biologics correlates. In the future, we will consider genomic characteristics associated with HCC prognosis, such as Ki-67 index [39], CK-19 [40], and p53 [41], to establish a more comprehensive radio-genomics model.

## Conclusion

In conclusion, the multi-sequence-based multiparametric radiomics nomogram for gadoxetic acid-enhanced MR imaging demonstrated good discriminative ability in predicting postoperative early recurrence (≤1 year) for HCC. Therefore, it may assist in postoperative outcome estimation and guide clinical treatment decision-making for patients with HCC.

## Additional file

Additional file 1 **Table S1**. MRI sequences and parameters. “Detailed MR imaging sequences and parameters are provided in Table S1.” **Table S2**. Selected radiomics features and their coefficients. “In total, 13 radiomics features with non-zero coefficients (2 features from T2-weighted images, 6 features from arterial phase images and 5 features from HBP images) were selected to calculate the radiomics score using the formula described in Table S2.” Part 2b. Detailed name and description of the selected radiomics features. “The name and description of the selected features can be found in Part 2b.” Part 3. The R software packages used for statistical analysis. “The packages in R that were used in this study are listed in Part 3.” (DOCX 120 kb)

## Abbreviations

AFP: Alpha-fetoprotein
ALB: Albumin
ALT: Alanine aminotransferase
AST: Aspartate aminotransferase
AUC: Area under the curve
CT: Computed tomography
DBIL: Direct bilirubin
DCA: Decision curve analysis
Gd-EOB-DTPA: Gadoxetic acid
GLCM: Grey-level co-occurrence matrix
GLRLM: Grey-level run-length matrix
HBP: Hepatobiliary phase
HBsAg: Hepatitis B virus surface antigen
HBV-DNA: Hepatitis B virus DNA load
HCC: Hepatocellular carcinoma
IBIL: Indirect bilirubin
ICC: Interclass correlation coefficient
LASSO: Least absolute shrinkage and selection operator
MRI: Magnetic resonance imaging
PLT: Platelet
PT: Prothrombin time
ROC: Receiver operating curve
ROI: Region of interest
TBIL: Total bilirubin
