# Supplementary material for: Hepatocellular carcinoma: radiomics nomogram on gadoxetic acid-enhanced MR imaging for early postoperative recurrence prediction
Source: Cancer Imaging. 2019 May 14;19:22. doi: 10.1186/s40644-019-0209-5 (PMC6518803; doi:10.1186/s40644-019-0209-5)
Supplement: Supplementary file 1 — Table S1. MRI sequences and parameters. “Detailed MR imaging sequences and parameters are provided in Table S1.” Table S2. Selected radiomics features and their coefficients. “In total, 13 radiomics features with non-zero coefficients (2 features from T2-weighted images, 6 features from arterial phase images and 5 features from HBP images) were selected to calculate the radiomics score using the formula described in Table S2.” Part 2b. Detailed name and description of the selected radiomics features. “The name and description of the selected features can be found in Part 2b.” Part 3. The R software packages used for statistical analysis. “The packages in R that were used in this study are listed in Part 3.” (DOCX 120 kb) [file 40644_2019_209_MOESM1_ESM.docx]

**Additional file 1**

***Part 1. The MR sequences and parameters information***

The MR sequences included: a breath-hold fat-suppressed fast spin-echo T2-weighted imaging, a MR cholangiopancreatography (MRCP) heavily T2-weighted 2D imaging, an in- and opposed-phase gradient-echo T1-weighted sequence, a diffusion-weighted sequence (b values: 0, 50, 500, 800, 1000, and 1200s/mm2), and a fat-suppressed 3D gradient-echo T1 weighted sequence (volume interpolated breath-hold examination, VIBE) before and after intravenous injection of Gd-EOB-DTPA at the arterial (bolus triggering, 7s after the signal intensity of the celiac trunk was the highest), portal venous (60-70s), transitional (3 min) and hepatobiliary phase (HBP, 20min). The acquisition protocols were shown in *Table 1*.

***Table 1*.** MRI sequences and parameters

| Sequence | Fat suppression | TR (ms) | TE (ms) | Flip angle | Section thickness (mm) | Matrix size | Field of view (mm^2^) | Acquisition time (s) |
| --- | --- | --- | --- | --- | --- | --- | --- | --- |
| T2-weighted 2D TSE | Used | 2160 | 100 | 160° | 6 | 320×288 | 433×433 | 36 |
| Coronal T2-weighted HASTE | Used | 1000 | 96 | 129° | 3 | 320×320 | 400×400 | 25 |
| T1-weighted 3D GRE VIBE | Used | 3,95 | 1.92 | 9° | 2 | 352×256 | 400×296 | 14 |
| T1-weighted IP and OP imaging | None | 81 | 1.4 | 70° | 6 | 352×286 | 400×325 | 24 |
| DW single-shot spin-echo EPI | Used | 5600 | 68 | 90° | 6 | 100×76 | 380×289 | 233 |
| MRCP T2-weighted HASTE | Used | 4500 | 709 | 180° | 40 | 384×269 | 300×300 | 4 |

*TR*, repetition time; *TE*, echo time; *2D*, two-dimensional; *3D*, three-dimensional; *TSE*, turbo spin-echo; *HASTE*, half fuorier single-shot turk spine-echo; *GRE*, gradient recall echo; *VIBE*, volume interpolated breath-hold examination; *IP*, in-phase; *OP*, opposed-phase; *DW*, diffusion-weighted; *EPI*, echo planar imaging; *MRCP*, magnetic resonance cholangiopancreatography.

***Part 2a. The calculation formula for radiomics score***

13 features were selected by using the least absolute shrinkage and selection operator (LASSO) method and a radiomics score was built for each patient through a linear combination of the selected features weighted by their coefficients (as described below). These features were from three categories: three were histogram based, one was texture based, seven were GLCM based, and two were GLRLM based.

***Table 2*.** Selected radiomics features and their coeffcients

| Sequence | Variables | Coefficients |
| --- | --- | --- |
| T2WI | (intercept) | 0.313146856623013 |
|  | Correlation_angle45_offset7 | 28.9891296416781 |
|  | Inertia_angle45_offset7 | -0.000279545503224346 |
| AP | (intercept) | -0.235519635140549 |
|  | Voxel Value Sum | 1.22885739390885E-08 |
|  | Skewness | 0.371490509266454 |
|  | Correlation_angle0_offset4 | 75.6065657339836 |
|  | Correlation_angle90_offset7 | 77.1256389958006 |
|  | Inertia_AllDirection_offset7_SD | -1.78418841775634E-06 |
|  | High Grey Level Run Emphasis_AllDirection_offset1_SD | -0.0000164468609913258 |
| HBP | (intercept) | 0.582580079480706 |
|  | Min Intensity | -0.00406625501505711 |
|  | Cluster Prominence_angle135_offset7 | 1.27250339620709E-08 |
|  | GLCMEnergy_angle45_offset7 | -18.0439367486555 |
|  | Inverse Difference Moment_AllDirection_offset4_SD | -802.586287878263 |
|  | High Grey Level Run Emphasis_AllDirection_offset4_SD | -0.000379815934092436 |

*T2WI*, T2-weighted imaging; *AP*, Arterial phase; *HBP*, hepatobiliary phase

*T2WI score* = 0.313146856623013 x (28.9891296416781 x Correlation_angle45_offset7 -0.000279545503224346 x Inertia_angle45_offset7)

*AP score* = -0.235519635140549 x (1.22885739390885E-08 x Voxel Value Sum + 0.371490509266454 x Skewness + 75.6065657339836 x Correlation_angle0_offset4 + 77.1256389958006 x Correlation_angle90_offset7 -1.78418841775634E-06 x High Grey Level Run Emphasis_AllDirection_offset1_SD)

*HBP score* = 0.582580079480706 x (-0.00406625501505711 x Min Intensity + 1.27250339620709E-08 x Cluster Prominence_angle135_offset7 -18.0439367486555 x GLCMEnergy_angle45_offset7 -802.586287878263 x Inverse Difference Moment_AllDirection_offset4_SD -0.000379815934092436 x High Grey Level Run Emphasis_AllDirection_offset4_SD)

*The radiomics score* = 0.278+1.2820 x T2WI score +2.1147 x AP score +1.3572 x HBP score

***Part 2b. Detailed name and description of the selected features are as follows:***

***Histogram***

Histogram parameters are concerned with properties of individual pixels. They describe the distribution of voxel intensities within the images through commonly used and basic metrics. Let X denote that three dimensional image matrix with N voxels and P the first order histogram divided by N discrete intensity levels.

1. **Skewness**

Skewness represents the degree of asymmetric distribution in the image histogram. High values of Skewness means that the distribution is asymmetric otherwise the image is more symmetric; negative skew is when the numerical distribution is relatively long also called negative Skewness distribution, the opposite is referred as positive Skewness distribution (positive skew).


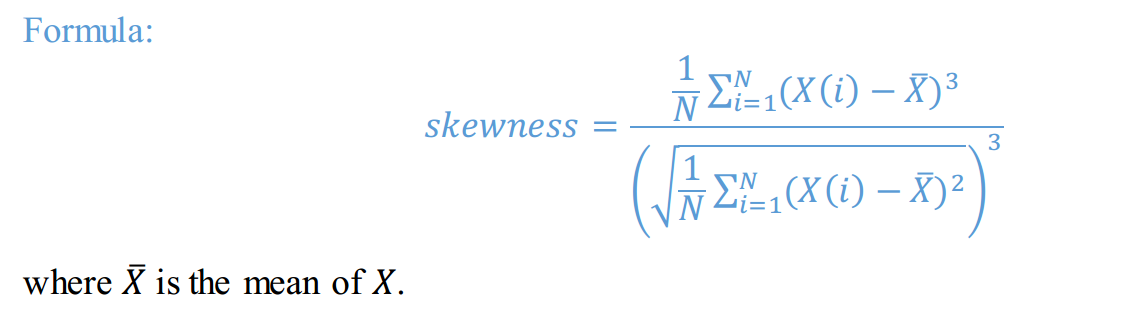


1. **Minintensity**

The minimum intensity value of X.

1. **Voxel Value Sum**

Voxel Value Sum represents the Sum calculations for voxels in the ROI.

***Texture***

Texture is one of the important characteristics used in identifying objects or regions of interest in an image, texture represents the appearance of the surface and how its elements are distributed. It is considered an important concept in machine vision, in a sense it assists in predicting the feeling of the surface (e.g. smoothness, coarseness …etc.) from image.

1. **Cluster Prominence_angle135_offset7**

Cluster Prominence is a measure of asymmetry of a given distribution, high values of this feature indicate that the symmetry of the image is low, in medical imaging low values of cluster prominence represent a smaller peak for the image grey level value and usually the grey level difference between the forms is small.


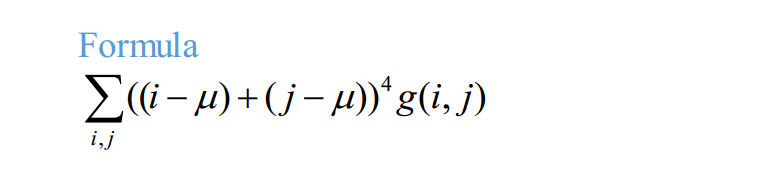


***Grey level co-occurrence matrix (GLCM)***

The Grey level co-occurrence matrix (GLCM) represents the joint probability of certain sets of pixels having certain grey-level values. It calculates how many times a pixel with grey-level **i** occurs jointly with another pixel having a grey value **j**. By varying the displacement vector **d** between each pair of pixels.

The rotation angle of an offset: 0°, 45°, 90°, 135° and displacement vectors (distance to the neighbor pixel: 1, 2, 3 ...), different co-occurrence distributions from the same image of reference.

1. **Correlation**

Correlation measures the similarity of the grey levels in neighboring pixels, tells how correlated a pixel is to its neighbor over the whole image. Range = [-1 1]. Correlation is 1 or -1 for a perfectly positively or negatively correlated image.

We have the 18 parameters related to the Correlation, including **Correlation_angle45_offset7**, **Correlation_angle0_offset4**, **Correlation_angle90_offset7**.

1. **Inertia of GLCM**

It reflects the clarity of the image and texture groove depth. The contrast is proportional to the texture groove, high values of the groove produces more clarity, in contrast small values of the groove will result in small contrast and fuzzy image.


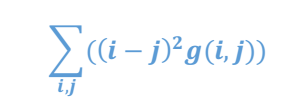


In AK Software we have the 18 parameters related to the Inertia, including **Inertia_AllDirection_offset7_SD and** **Inertia_angle45_offset7**

1. **GLCMEnergy_angle45_offset7**

This feature Returns the sum of squared elements in the GLCM. Range = [0 1]
Energy is 1 for a constant image. Is high when image has very good homogeneity
or when pixels are very similar. The property energy is known as uniformity, uniformity of energy, and angular second moment.


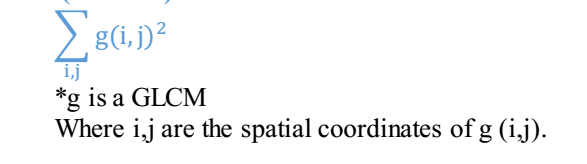


1. **Inverse Difference Moment_AllDirection_offset4_SD**

Inverse Difference Moment (IDM) is the local homogeneity. It is high when local gray level is uniform and inverse GLCM is high. IDM weight value is the inverse of the Contrast weight.


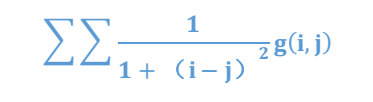


***Grey level run-length matrix (RLM)***

The grey level run-length matrix (RLM) is defined as the numbers of runs with
pixels of gray level *i* and run length *j* for a given direction θ. RLMs is generated for each sample image segment having directions (0°,45°,90° &135°), then the following statistical features.

1. **High Grey Level Run Emphasis_AllDirection_offset1_SD**


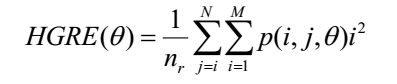


1. **High Grey Level Run Emphasis_AllDirection_offset4_SD**


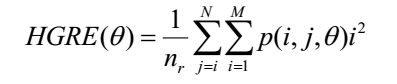


***Part 3. The R software packages used for statistical analysis***

R software version 3.2.3 was used in this study. The packages we used in this study are described as following.

1. LASSO logistic regression was performed using the “glmnet” package.
2. The “pROC” package was used for the ROC curve analysis.
3. Nomogram construction were performed within the “rms” package.
4. The Hosmer-Lemeshow test and calibration plots was done with the “nricens&PredictABEL” package.
5. DCA was performed with the function “rmda” package.
